# Elevated Positive End-Expiratory Pressure Decreases Cardiac Index in a Rhesus Monkey Model

**DOI:** 10.3389/fped.2014.00134

**Published:** 2014-12-03

**Authors:** Patrick A. Ross, Robinder G. Khemani, Sarah S. Rubin, Anoopindar K. Bhalla, Christopher J. L. Newth

**Affiliations:** ^1^Children’s Hospital Los Angeles, University of Southern California Keck School of Medicine, Los Angeles, CA, USA

**Keywords:** positive end-expiratory pressure, cardiac index, oxygen transport, *Macaca mulatta*

## Abstract

**Rationale:** Clinicians are often concerned that higher positive end-expiratory pressure (PEEP) will decrease cardiac index (CI). PEEP affects CI through multiple inter-related mechanisms. The adult Rhesus monkey is an excellent model to study cardiopulmonary interaction due to similar pulmonary and chest wall compliances to human infants.

**Objective:** Our goal was to examine the impact of increasing PEEP on CI in Rhesus monkeys as a model for critically ill children.

**Methods:** Prospective, experimental animal study. Nine healthy anesthetized, intubated Rhesus monkeys were allowed to breathe spontaneously at a PEEP of 0, 5, 10, and 15 cm H_2_O while CI was measured with an ultrasonic Doppler (USCOM).

**Measurements and main results:** Cardiac index decreased between PEEP levels of 5 and 15 cm H_2_O. The mean decrease in CI for the entire cohort of monkeys was 18% (*p* < 0.01) with a range of −11 to 49%. Stroke volume and oxygen delivery also decreased between PEEP levels of 5 and 15 cm H_2_O (*p* < 0.01).

**Conclusion:** Between PEEP levels of 5 and 15 cm H_2_O, there was a decrease in CI, stroke volume, and oxygen delivery in intubated Rhesus monkeys. A plausible mechanism is that over-distention of normally compliant lungs at increased PEEP resulted in decreased preload to the right ventricle, outweighing the potentially beneficial decrease in left ventricular afterload or pulmonary vascular resistance. Further investigation is warranted, particularly in children with lung injury, who have historically benefited from increased PEEP levels without over-distention.

## Introduction

Clinicians are often concerned that higher positive end-expiratory pressure (PEEP) levels needed to achieve lung recruitment in patients with acute lung injury (ALI) will decrease cardiac index (CI) and potentially decrease oxygen delivery. There are limited studies (1, 2) on the effect of PEEP on CI in children due to the perceived risk of invasive techniques required for direct CI measurement in the past. Recently, minimally invasive techniques have been developed, which offer a safe method of obtaining CI measurements in children (3, 4). We used ultrasound Doppler to investigate the effect of PEEP on CI, stroke volume, and oxygen delivery in a controlled environment using a Rhesus monkey model that has significant physiological similarities to children.

Positive end-expiratory pressure affects CI through multiple inter-related mechanisms (5, 6). On one hand, PEEP may decrease CI by increasing intrathoracic pressure and decreasing preload to the right ventricle. On the other, PEEP may increase CI by decreasing left ventricular afterload and if there is atelectasis by lung recruitment leading to decreased pulmonary vascular resistance and right ventricular afterload. However, as PEEP is increased further there may be over-distention of the airways, increasing pulmonary vascular resistance causing increased right ventricular afterload resulting in decreasing CI. This complex physiological relationship may vary based on the baseline myocardial function, intravascular volume, and compliance of the lungs. Decreased cardiac output with increasing tidal volumes and pulmonary over-distention has previously been demonstrated in a piglet swine model (7). However, PEEP was not increased >10 cm H_2_O in the piglet model. Further, similar pulmonary and chest wall compliances cannot be assumed between the swine and human infant.

Adult Rhesus monkeys have proven to be an excellent surrogate for human infants in respiratory mechanics and cardiopulmonary interaction research due to similarities in respiratory (8, 9) and cardiovascular (10) physiology. The Rhesus monkey model allows studies to be performed in a controlled setting. Our primary outcome measure was change in CI at increasing levels of PEEP. The effects of PEEP on secondary outcome measures such as vital signs and oxygen delivery were also considered. Given results from prior studies (4, 7), we hypothesized that increased PEEP will decrease CI in intubated, spontaneously breathing, Rhesus monkeys with normal respiratory system compliance.

## Materials and Methods

We performed an interventional study in adult Rhesus monkeys that were of similar weight and pulmonary development to human infants. The Cantonal Animal Protection Committee in Basel, Switzerland and the Institutional Animal Care and Use Committee (IACUC) at Children’s Hospital Los Angeles approved this study. The study was conducted at the Novartis Animal Care Facility in Basel, Switzerland. Prior routine pulmonary function tests on this cohort of animals demonstrated normal respiratory compliance (0.8–1.1 ml/cm H_2_O). There was no clinical evidence of cardiac disease among the monkeys. A convenience sample of nine monkeys was available during the time allotted for study. We induced anesthesia in the monkeys with 100 mg of intramuscular ketamine. After sedation had taken effect, we inserted a 20 gauge peripheral IV and obtained hemoglobin measurements for calculation of oxygen delivery. Oxygen delivery was calculated as (cardiac output)*(1.34*hemoglobin*oxygen saturation) without measurement of PaO_2_. PaO_2_ was not included as we did not obtain arterial blood gas measurements and contribution of PaO_2_ is likely negligible in monkeys with normal hemoglobin levels. A propofol infusion was started at 10 mg/kg/h and titrated to achieve an appropriate level of sedation while allowing the monkeys to breathe spontaneously. We performed direct laryngoscopy and sprayed the vocal cords with 1% topical lidocaine. We intubated the monkeys with a 4.5-cuffed endotracheal tube (Rüsch, Research Triangle Park, NC, USA) and inflated the cuff to occlude any audible air leak. We continually monitored heart rate, respiratory rate, temperature, and peripheral arterial oxygen saturations. We measured non-invasive cuff blood pressure every 5 min or more as clinically indicated. We placed a pneumotachometer calibrated to zero flow (Bicore II, Carefusion, Houten, The Netherlands) in line between the endotracheal tube and anesthesia bag. We maintained PEEP at levels of 0, 5, 10, and 15 cm H_2_O using a Jackson–Reese modification to a flow inflating anesthesia bag with an inline manometer. The upper limit of PEEP was chosen as 15 cm H_2_O based on clinical experience in critically ill children. Further previous studies have suggested that in the routine care of mechanically ventilated children PEEP is rarely increased above 12 cm H_2_O (11). The animals were breathing room air spontaneously without additional pressure support. We maintained PEEP at each level for 5 min and measured CI during the last 2 min. The Rhesus monkeys were allowed to return to a baseline of 0 PEEP between each intervention. Two investigators measured CI and the first 20 measurements were obtained by both investigators to demonstrate inter-rater reliability.

The Ultrasonic Cardiac Output Monitor (USCOM-1A, USCOM Pty Ltd, Coffs Harbor, NSW, Australia) was introduced for clinical use in 2001. CI measured by USCOM correlates favorably with CI measured by thermodilution (12, 13). USCOM uses a Doppler ultrasound probe in the aortic notch to measure the velocity of blood flow through the aortic valve. The velocity of blood is multiplied by the cross-sectional area of the valve to determine stroke volume. The stroke volume is multiplied by heart rate to determine cardiac output, which we divided by body surface area to produce CI. The USCOM software uses entered blood pressures to calculate systemic vascular resistance. We obtained two hemodynamic measurements for each monkey at each PEEP level. All monkeys completed the protocol without harm and were returned to normal care in the animal facility.

### Statistical analysis

The primary outcome measure was a change in CI at increasing levels of PEEP. Mean difference in CI, hemodynamic variables, and vital signs at different levels of PEEP were analyzed using Repeated Measures Analysis of Variance (ANOVA). If a difference was seen with ANOVA, then a Bonferroni *post hoc* test was performed to allow for multiple comparisons between levels of PEEP. Non-parametric data were log or square root transformed to achieve normality for ANOVA analyses. Plots of CI at each level of PEEP were generated for the group and for each individual monkey. Average bias and 95% limits of agreement were calculated on duplicate measurements to demonstrate inter-rater agreement.

### Funding

The Novartis Pharmaceuticals Corporation provided use of the animal care facilities and Rhesus monkeys for the research study. No additional funding was provided by Novartis or any other source.

## Results

**Figure 1:**
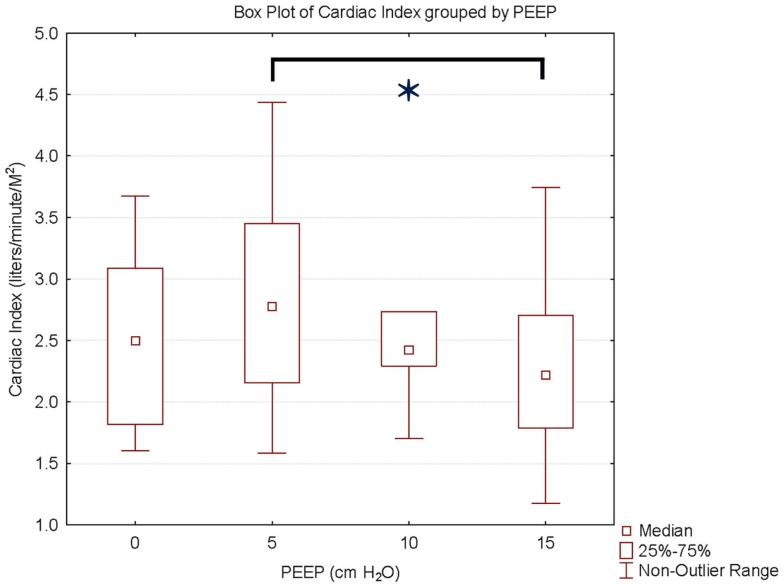
**Cardiac index and PEEP**. Data are presented as median, inter-quartile, and non-outlier range. There is a statistically significant decrease in cardiac index at PEEP of 15 cm H_2_O compared to PEEP 5 cm H_2_O.

**Figure 2:**
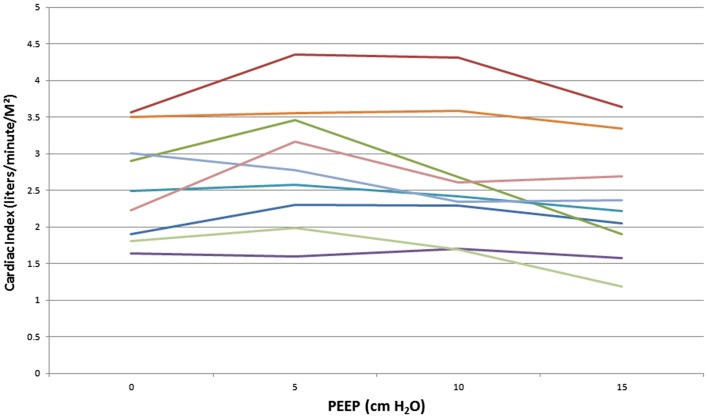
**Cardiac index at increasing PEEP levels for individual monkeys**.

We studied nine Rhesus monkeys with the following characteristics: weight: median, IQR = 15.3 kg (13.5, 16.2); height: median, IQR = 88 cm (84, 92); hemoglobin: median, IQR = 15.2 g/dl (14.7, 15.6). All monkeys tolerated the study without complications. Measurements between the two investigators were examined to determine inter-rater reliability. The difference between CI measurements showed a bias of −0.02 l/min/M^2^ with 95% limits of agreement of (−0.4 to 0.4 L/min/M^2^). Hemodynamic measurements at increasing PEEP are shown in Table 1. There was a statistically significant decrease in CI, stroke volume, and oxygen delivery between PEEP levels of 5 and 15 cm H_2_O. The mean decrease in CI for the entire cohort of monkeys was 18% (*p* < 0.01) (Figure 1) with a range of −11 to 49% (Figure 2). CI, stroke volume, and oxygen consumption were not significantly different between PEEP levels of 5 and 10 cm H_2_O and 10 and 15 cm H_2_O. There was no significant change in systemic vascular resistance between the various PEEP levels.

**Table 1:** **Hemodynamic measurements during increasing positive-end expiratory pressure**.

PEEP (cm H_2_O)	0	5	10	15
Cardiac index (l/min/m^2^)	2.5 (1.9, 3.0)	2.8 (2.3, 2.7)	2.4 (2.3, 2.7)	2.2 (1.9, 2.7)^†^
Stroke volume (cm^3^)	13.1 (9.1, 13.3)	13.5 (10.5, 14.5)	11.3 (10.3, 13.0)	10.7 (8.4, 13.3)^†^
Systemic vascular resistance (dynes∙s/cm^5^)	3791 (3570, 6138)	4006 (3381, 4985)	4503 (3976, 6351)	4315 (3558, 5521)
Oxygen delivery (l/min)	43.6 (36.4, 56.2)	46.7 (43.0, 58.8)	42.6 (29.4, 52.1)	39.7 (27.7, 49.0)^†^

*Measurements are presented as median (inter-quartile range)*.

*^†^Significantly different as compared to PEEP 5 cm H_2_O, *p* < 0.01*.

*All other comparisons were not statistically significant*.

Oxygen delivery = (cardiac output)*(1.34*hemoglobin*oxygen saturation).

Vital signs with increasing PEEP are shown in Table 2. There was no significant difference in heart rate, systolic blood pressure, diastolic blood pressure, or end tidal carbon dioxide between the various PEEP levels. There was a statistically significant increase in tidal volume between a PEEP of 0 cm H_2_O and all other PEEP values. Tidal volume did not change significantly between PEEP levels of 5, 10, and 15 cm H_2_O. There was a significant decrease in respiratory rate among PEEP levels of 0, 10, and 15 cm H_2_O. There was also a significant decrease in respiratory rate between PEEP levels of 5 and 15 cm H_2_O. Minute ventilation at a PEEP of 5 cm H_2_O was significantly greater than all other PEEP levels.

**Table 2:** **Vital signs measured during increasing positive end-expiratory pressure**.

PEEP cm H_2_O	0	5	10	15
Heart Rate (bpm)	137 (134, 151)	143 (138, 153)	141 (135, 154)	143 (122, 156)
Systolic BP (mmHg)	131 (120, 142)	152 (134, 158)	156 (136, 165)	144 (107, 156)
Diastolic BP (mmHg)	67 (63, 85)	76 (73, 84)	82 (73, 90)	77 (56, 84)
Respiratory rate (per min)	42 (38, 46)	36 (28, 43)	28 (22, 35)*^,^****	27 (19, 31)*^,^****
Tidal volume (ml)	45 (44, 48)	57 (50, 65)*	72 (53, 80)*	68 (56, 78)*
Minute ventilation (l/min)	1.9 (1.6, 2.0)	2.1 (1.8, 2.4)**	1.9 (1.4, 2.2)	1.7 (1.3, 1.8)
End tidal CO_2_ (mmHg)	43 (43, 44)	46 (44, 46)	44 (41, 45)	43 (41, 47)
Oxygen saturations (%)	88 (83, 88)	86 (79, 87)	84 (78, 88)	81 (73, 88)***
Temperature (°C)	37.9 (37.0, 38.6)	37.8 (36.6, 38.2)*	37.7 (36.9, 38.1)*	37.7 (36.9, 38.1)*

*Measurements are presented as median (inter-quartile range)*.

*Each significantly different as compared to PEEP 0 cm H_2_O, *p* < 0.02.

**Significantly different as compared to PEEP 0 and 15 cm H_2_O, *p* < 0.05.

***Significantly different as compared to PEEP 0 cm H_2_O, *p* < 0.05

****Significantly different as compared to PEEP 5 cm H_2_O, *p* < 0.05.

*All other comparisons were not statistically significant*.

## Discussion

We found a statistically significant decrease in CI between PEEP levels of 5 and 15 cm H_2_O in Rhesus monkeys. The mean decrease in CI was 18% between these PEEP levels. However, as was shown in Figure 2, the decrease in CI appears to have been due to changes in just a few animals. Between the PEEP levels of 0 and 15 cm H_2_O, two animals had a decrease in CI of 35%, and one animal had a decrease in CI of 22%. Stroke volume was significantly decreased at higher PEEP levels. This suggests that increased intrathoracic pressure caused by higher PEEP levels may decrease preload to the right ventricle, outweighing the potentially beneficial decrease in left ventricular afterload or pulmonary vascular resistance. This effect may have been more pronounced in a few animals due to differences in their intravascular volume with resultant greater variation on the venous return curve. As there was no significant change in systemic vascular resistance, it does not appear that vascular tone was changed during our measurements.

Ongoing research on mechanical ventilation strategies in children provides insight into possible mechanisms for the effects of PEEP on CI. In restrictive lung disease, PEEP expands the lungs closer to functional residual capacity (FRC) to reduce secondary injury to the lungs from atelectotrauma and decreases the need for high FiO_2_ (14). Studies have shown that a high PEEP, low tidal volume mechanical ventilation strategy can reduce mortality in adults with acute respiratory distress syndrome (ARDS) and ALI (15, 16). However, clinicians, particularly pediatricians, often choose to limit PEEP and use high FiO_2_ when ventilating patients with ALI (11, 17). One reason for this is concern about the negative effects of PEEP levels >12 cm H_2_O on CI. In our study, the median decrease in CI between PEEP levels of 10 and 15 cm H_2_O was 8%. In addition, a clinically significant decrease in CI between these PEEP levels only occurred in two monkeys.

Between PEEP levels of 0 and 5 cm H_2_O, there was an increase in CI, stroke volume, and oxygen delivery. These values were not statistically significant but may be clinically relevant. It is possible that they reflect positive pressure reversing atelectasis that occurred during intubation.

We noticed a significant decrease in oxygen delivery at higher PEEP levels. We believe that this was due to a decrease in CI in combination with low baseline oxygen saturations. While the Rhesus monkeys in this cohort have lower baseline oxygen saturations than we have previously seen from animals in this colony, their compliance was normal (8). We believe changes in ventilation/perfusion matching or diffusion block may have affected peripheral oxygen saturations in older monkeys. Applying high levels of PEEP to normally compliant lungs may have caused over-distention of the lungs in some monkeys resulting in decreased pulmonary blood flow and decreased CI or worsening of intrapulmonary shunt leading to the decreased oxygen saturations we observed.

We noticed a statistically significant decrease in temperature during the study. However, we believe that the change in temperature (0.4°C maximum) was small enough to be of no clinical relevance.

Our study should be viewed in the context of its limitations. We did not create an animal model of lung injury as it would entail additional risks to the monkeys. There were a relatively small number of monkeys available for this study. As opposed to other animal studies (7), there was variability in the monkey’s response to PEEP. This may represent the age or condition of the various monkeys. The monkeys were breathing spontaneously on CPAP, which may alter venous return and therefore CI. Finally, the aortic valve diameter used for calculations with the USCOM measurements was obtained from a human primate nomogram (18). The aortic valve areas generated from the nomograph for the monkeys ranged from 1.08 to 1.21 cm, which is consistent with values obtained by other investigators (10, 19). Further, the aortic valve area would remain constant for each monkey during the study enabling accurate comparison of percent change in CI. Despite these limitations, we believe that given the respiratory and cardiac similarities to infants, the Rhesus monkey remains a good surrogate for these types of studies.

Our findings are consistent with previous animal research by Cheifetz et al. (7) who showed a decrease in cardiac output with increasing PEEP and increasing tidal volumes in a piglet model. At a baseline tidal volume of 10 ml/kg, they showed a 30% reduction in cardiac output when PEEP was increased from 5 to 10 cm H_2_O. There are a few key differences between our methods. The goal of their study was to demonstrate the effect of lung over-distension and in turn the piglets received muscle relaxation and minute ventilation as well as tidal volume was controlled. In our study, the Rhesus monkeys were allowed to breathe spontaneously on PEEP with no control over minute ventilation or tidal volume. The effect of higher PEEP levels on CI was less in our study (18% compared to 30%) and may be the result of the physiological differences associated with spontaneous ventilation as compared to controlled mechanical ventilation.

Our findings are also consistent with previous pediatric studies reporting a statistically significant decrease in CI with increased PEEP up to 12 cm H_2_O (20). These studies were performed in children with normal or near-normal respiratory system compliance that were near the end of their course of mechanical ventilation. These findings further confirm the suitability of the Rhesus monkey model for investigations of infants and children (who have similar cardiopulmonary physiology). Furthermore, these studies report that there were no notable clinical consequences of decreased CI suggesting that the negative cardiac effects of increased PEEP may not be clinically relevant. Additional work is needed to determine the effect of PEEP in children with significant lung injury and cardiac impairment.

## Conclusion

Between PEEP levels of 5 and 15 cm H_2_O, there was a decrease in CI, stroke volume, and oxygen delivery in intubated Rhesus monkeys with normal respiratory system compliance. Potentially over-distention of normally compliant lungs at increased levels of PEEP may have resulted in a decrease in preload to the right ventricle, without the potentially beneficial decrease in left ventricular afterload. Further investigation is warranted, particularly in children with lung injury, who may be less at risk for over-distention with PEEP levels of 15 cm H_2_O.

## Conflict of Interest Statement

The authors declare that the research was conducted in the absence of any commercial or financial relationships that could be construed as a potential conflict of interest.

## Abbreviations

CI, cardiac index; PEEP, positive end-expiratory pressure; SpO_2_, oxygen saturations by pulse oximetry.
